# Anodal transcranial direct current stimulation increases corticospinal excitability, while performance is unchanged

**DOI:** 10.1371/journal.pone.0254888

**Published:** 2021-07-16

**Authors:** Mathias Kristiansen, Mikkel Jacobi Thomsen, Jens Nørgaard, Jon Aaes, Dennis Knudsen, Michael Voigt

**Affiliations:** Department of Health Science and Technology, Sport Sciences–Performance and Technology, Aalborg University, Aalborg, Denmark; University Medical Center Goettingen, GERMANY

## Abstract

Anodal transcranial direct current stimulation (a-tDCS) has been shown to improve bicycle time to fatigue (TTF) tasks at 70–80% of VO_2max_ and downregulate rate of perceived exertion (RPE). This study aimed to investigate the effect of a-tDCS on a RPE-clamp test, a 250-kJ time trial (TT) and motor evoked potentials (MEP). Twenty participants volunteered for three trials; control, sham stimulation and a-tDCS. Transcranial magnetic stimulation was used to determine the corticospinal excitability for 12 participants pre and post sham stimulation and a-tDCS. The a-tDCS protocol consisted of 13 minutes of stimulation (2 mA) with the anode placed above the Cz. The RPE-clamp test consisted of 5 minutes ergometer bicycling at an RPE of 13 on the Borg scale, and the TT consisted of a 250 kJ (∼10 km) long bicycle ergometer test. During each test, power output, heart rate and oxygen consumption was measured, while RPE was evaluated. MEPs increased significantly by 36% (±36%) post a-tDCS, with 8.8% (±31%) post sham stimulation (*p* = 0.037). No significant changes were found for any parameter at the RPE-clamp or TT. The lack of improvement may be due to RPE being more controlled by afferent feedback during TT tests than during TTF tests. Based on the results of the present study, it is concluded that a-tDCS applied over Cz, does not enhance self-paced cycling performance.

## Introduction

Transcranial direct current stimulation (tDCS) is a non-invasive brain stimulation technique, applied as a constant low-intensity current (1–2 mA) on the scalp through saline-saturated sponge electrodes [1]. Transcranial direct current stimulation has been shown to modulate the corticospinal excitability in a polarity-dependent manner. More specifically, positive stimulation known as anodal tDCS (a-tDCS) increases corticospinal excitability, while negative stimulation known as cathodal tDCS decreases corticospinal excitability [1,2].

Recently, a-tDCS has been investigated as a potential ergogenic aid in sports performance. For instance, in isometric time-to-fatigue (TTF) tasks, a-tDCS has been shown to improve performance of healthy participants in both the upper [3–6] and lower extremities [7]. Moreover, a-tDCS has been shown to improve TTF during cycling tasks at 70% and 80% of VO_2max_ [8–10] as well as during an incremental cycling test to failure [11]. In these studies the improved performances were associated with a decrease in rating of perceived exertion (RPE) and an increase in corticospinal excitability [8,9,11,12]. Currently, the physiological mechanisms underlying possible performance enhancement of a-tDCS are not fully understood [13]. It has previously been hypothesized that an a-tDCS induced increase in excitability of M1 warrants less excitatory input from upstream cortical areas to produce the same motor output needed to activate the muscles at any given intensity, and thereby causes reductions in RPE [9,13]. Another possible mechanism involves the ability of a-tDCS to directly decrease the rate of perceived exertion during exercise. This has previously been reported when a-tDCS was applied before exercise, and could thus serve as an explanation to the observed performance enhancements [7,11].

Despite the performance improvements observed in TTF-tasks, it should be noted that this type of performance task may not adequately reflect the demands of real-world sports competition such as time trials (TT), where the primary aim is to complete a given distance in the shortest amount of time [14,15]. To the best of our knowledge only one study has investigated the influence of a-tDCS on TT performance during cycling [14]. However, no difference in performance between a-tDCS and sham was found [14]. This lack of performance improvement could be due to a methodological choice, as the cathodal electrode was placed over the contralateral supraorbital area, potentially leading to cathodal stimulation of the dorsolateral prefrontal cortex [7], which has previously been shown to negate the positive effects of a-tDCS on endurance performance [7]. Thus, improved performance during a cycling TT could still be possible using the recommended placement on the contralateral shoulder [7].

To achieve the shortest time possible in competition, such as in a TT, athletes rely on pacing strategies [16]. A pacing strategy is defined as the regulation of energetic resources to maintain sustainable intensity throughout the exercise and to avoid premature fatigue and exhaustion [16]. During self-paced exercise the mechanisms underlying the regulation of the pacing strategies are still widely unknown, however, some evidence points towards RPE being a key regulator [17]. Anodal-tDCS has previously been shown to decrease the RPE during TTF cycling exercise [9,11]. If the downregulation of RPE is similar during a TT, this could lead to an improved performance, due to an increased exercise tolerance and altered pacing strategy. Such information would further provide more ecological valid data for the use of a-tDCS as an ergogenic aid to real-world performance.

The aim of the present study was therefore to determine the effects of a-tDCS applied over M1 on corticospinal excitability and self-paced cycling performance during a submaximal RPE-clamp test and a TT. Based on previous findings of a-tDCS induced downregulation of RPE during TTF-tasks [8,9,11,12], we hypothesized that power output would increase during the RPE-clamp test, and that performance in a TT would be improved in the a-tDCS protocol compared to the sham stimulation protocol.

## Materials and methods

### Participants

The study was approved by the local Ethics Committee (VN 20170081) and carried out in accordance with the Declaration of Helsinki. Twenty-two healthy recreationally trained participants were recruited from February to April, 2018, and volunteered for this sham-controlled study. Two participants were excluded due to injuries obtained unrelated to the study. The remaining participants were all included in the final analysis and consisted of 16 males and four females that on average (± standard deviation) were: 26 (±4) years, 181.5 (±9.9) cm and 83.9 (±17.9) kg. Participants were excluded if they had any physical or neurological disorders that could affect their performance. All participants were given a detailed verbal and written explanation of the experimental risks before providing a written informed consent. Twelve of the participants also volunteered to receive transcranial magnetic stimulation (TMS) to assess changes in corticospinal excitability through recording of motor evoked potentials. These participants were screened prior to participation using the Transcranial Magnetic Stimulation Adult Safety Screen (TASS) [18]. If requirements from the TASS was not meet, participants were excluded. The number of participants included in the TMS protocol was calculated using an α-level, β-level, and effect size of 0.05, 0.8, and 1.2, respectively. Effect size estimation was based on the results of Bastani et al [19].

### Experimental protocol

**Fig 1:**
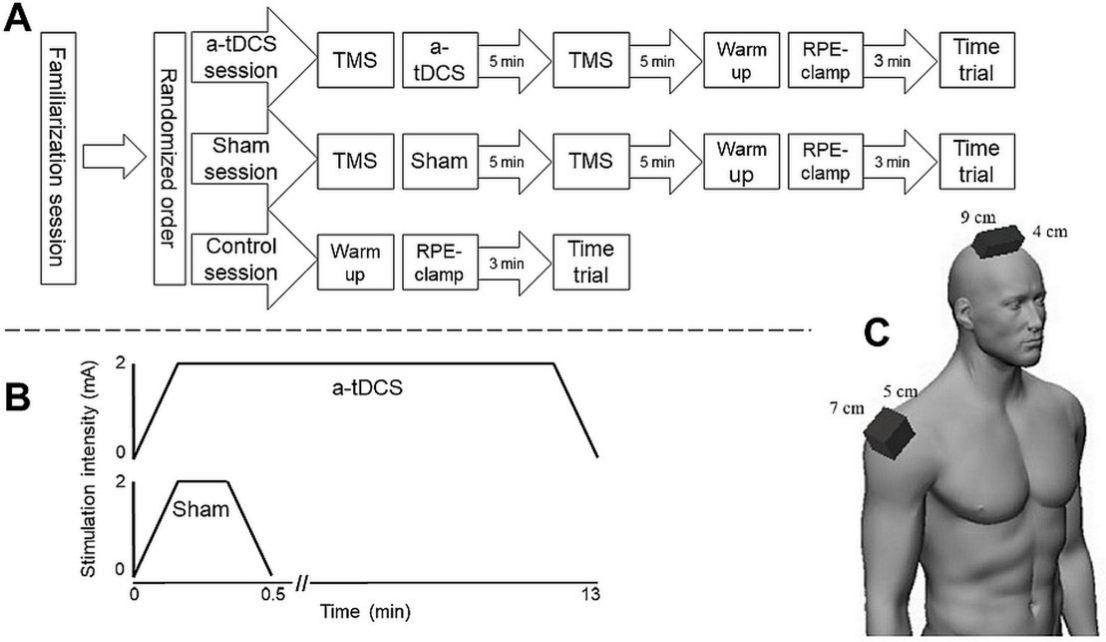
**A:** Illustrates the experimental design of the present study. **B:** Illustrates the applied stimulation protocol for a-tDCS and sham stimulation. **C.** Illustrates the placement of the electrodes, with the anode centered above the Cz, and the cathode centered above the right deltoid muscle. a-tDCS = anodal transcranial direct current stimulation. TMS = Transcranial Magnetic Stimulation (Twelve participants completed the TMS protocol). RPE = Rate of perceived exertion. mA = milliampere.

In the present study, participants completed one familiarization session, and three test sessions (Fig 1A). The three test sessions were: a control test session, a sham stimulation test session, and an a-tDCS test session. All sessions were separated by at least 48 hours and completed within 14 days in the period between February and June, 2018. The familiarization session was completed first followed by the remaining three test sessions, which were carried out in a counterbalanced order. At the familiarization session, the ergometry bike (Lode, Excalibur Sport, Groningen, The Netherlands) was adjusted to fit the individual, where after the participants were familiarized with the RPE-clamp test and the 250-kJ TT. On the a-tDCS and sham stimulation test days, the participants underwent superimposed single-pulse TMS, and MEPs were recorded from the Rectus Femoris (RF) muscle to assess corticospinal excitability. Then, the a-tDCS and sham stimulation protocols were carried out, and five minutes post stimulation the corticospinal excitability was assessed again. Ten minutes following the cessation of the stimulation protocol, the participants completed a three-minute warm-up, which was directly followed by an RPE-clamp test, where the participants were instructed to bike for five minutes with an RPE of 13 on the Borg scale [20]. Following three minutes of rest, participants then performed a 250-kJ TT. On the control test day, the RPE—clamp test and the 250kJ TT was also carried out, but no stimulation or MEP recording occurred. The control session was incorporated to control for any unsolicited responses brought about by the active sham stimulation procedures used in this study [21]. The design of the present study is illustrated in Fig 1A.

### Transcranial magnetic stimulation

For the 12 participants receiving TMS, the a-tDCS and sham test days started with surface electromyography (sEMG) electrodes being mounted on rectus femoris (RF) on the right leg. First, the skin was shaved, abraded, and cleaned with alcohol, before two surface electromyography electrodes (Ambu Neuroline 720 01-K/12, Ag/AgCl, inter electrode distance 20 mm, Ambu A/S, Ballerup, Denmark) were mounted parallel to the muscle fiber direction according to the SENIAM guidelines. A reference electrode was mounted between the two recording electrodes. A Magstim stimulator (Magstim 200, figure of eight coil shape, coil size 70mm, Magstim Company, Dyfed, UK) was used to determine the hotspot, resting motor threshold (RMT), and MEP amplitude of the RF. To determine the hotspot, three consecutive stimuli over Cz were delivered where after the coil was moved in ~1cm steps laterally, until the highest and most consistent MEPs were elicited. This position was marked on the scalp with a felt pen. Subsequently, RMT was identified, and defined as the minimal stimulus intensity at which 5 out of 10 consecutive stimuli evoked a MEP with an amplitude of at least 50 μV in the resting muscle Twenty MEPs were then elicited at 120% RMT stimulus intensity. MEP´s were recorded at two time points. Firstly, prior to the a-tDCS protocol and secondly five minutes post the stimulation protocol.

### Transcranial direct current stimulation

A battery driven, constant current stimulator (Linear Stimulus Isolator A395, World Precision Instruments, Sarasota, Florida, USA) delivered a-tDCS through two custom-made plate electrodes. Thin physiological saline saturated sponges were placed between the electrode and the skin. Impedance was kept below 5 kΩ. For the twelve participants who had received TMS, the anodal electrode (9 x 4 cm) was placed on top of the previously located hotspot of the RF muscle. For the remaining the participants, the anodal electrode was placed on top of Cz, as the electrode would then be certain to cover the hotspot of RF on both hemispheres, due to its size (9 x 4cm) [22]. The cathodal electrode (7 x 5 cm) was fixed at the center of the deltoideus medialis muscle on the right shoulder (Fig 1C). Stimulation intensity was set to 2 mA. The current was progressively increased during the first 10 seconds and then held constant for 13 minutes for the a-tDCS stimulation [8], where after it was progressively decreased for 10 seconds. During the sham stimulation a single blinded procedure was carried out, in which the current was progressively increased during the first 10 seconds, then held constant for 30 seconds, before it was progressively decreased for 10 seconds (Fig 1B).

### Bicycle tests

The Lode ergometry bike was set to linear mode (power output increases linearly with pedaling frequency). The power output (PO) was measured in Watt, and calculated through the formula

$$PO[W]=\alpha \times {\left(n[min^{-1}]\right)}^{2}$$

with *n* being revolutions per minute and α being the resistance. Sampling frequency was 50 Hz. Before each cycling test, participants had a three-minute self-paced warm-up with incremental resistance, on the ergometry bike. Three blocks of one minute was performed with an α resistance of 0.025, 0.034 and 0.042. The warm-up led directly to a five-minute RPE-clamp cycling test at a self-selected α resistance. The RPE-clamp test was used to evaluate the influence of a-tDCS on RPE during self-paced cycling, by comparing the PO between conditions. During the RPE-clamp test, participants were instructed to maintain a constant RPE of 13. Participants were given visual feedback on time spent and brake resistance during the test. Following the RPE-clamp test, participants performed a 250-kJ TT, previously estimated to reflect a distance of ∼10 km [23]. Participants were instructed to finish the TT as fast as possible. During the TT, no temporal or physiological feedback were given. Participants received visual feedback on accumulated kJ and verbal encouragement. The α resistance was set in accordance to individual preferences, based on the familiarization trial. The choice of using of a time trial test, as opposed to a constant work rate test, was done based on research showing that critical power and severe-intensity exercise performance is enhanced in time trial tests [24]. Participants could at any time inform the experimenter to increase or decrease the resistance in steps of 0.005. The protocol was identical for the control, sham stimulation, and a-tDCS test days.

### Physiological measurements

All of the physiological measurements were collected throughout the warm-up, RPE-clamp test, and TT. Heart rate (HR) was recorded by a HR monitor (Wearlink+transmitter, Polar Electro Oy, Finland). Pulmonary gas exchange was measured breath-by-breath using a computerized metabolic measurement system (Jaeger Vyntus CPX, CareFusion, Hoechberg, Germany) connected to a face-mask with a digital volume transducer. The system was calibrated prior to each test using standardized gas concentrations (15% O_2_ and 5% CO_2_). The software used to run the Jaeger Vyntus CPX (Version 2.19.96, SentrySuite, CareFusion, Hoechberg, Germany) automatically transformed the data into standard temperature (0°C) and pressure (760 Torr) [25].

### Rating of perceived exertion

Rating of perceived exertion was measured using a 15-grade Borg 6–20 scale [20]. The RPE scale was displayed in front of the participants throughout the entire test and participants were told to rate their RPE by pointing on the scale every 25 kJ of the TT and upon completion.

### Electromyographic recording

A custom made EMG amplifier (Aalborg University, Denmark) and a custom made LabVIEW©-based computer program (Mr. Kick© v2.030e, University of Aalborg, Aalborg, Denmark) was used to collect EMG signals at sampling rate of 5 KHz, with a sensitivity of 500 mV/V and using a 5–1000 Hz band pass filter.

### Data analysis

The size of the MEP response in each participant was defined as the average peak-to-peak value of the 20 MEPs recorded in either the a-tDCS or Sham stimulation session. The relative difference between pre and post stimulation MEP amplitude was calculated in percent for each participant and each session. PO (W), HR (beats/min) and VO_2_ (ml/min) signals were synchronized and averaged over a 1-minute epoch between the third and fourth minute of the RPE-clamp and for every 25-kJ completed of the TT, to coincide with RPE rating. Further, a ratio between PO/RPE was calculated for every 25-kJ epoch of the TT.

### Statistical analysis

To test for changes in MEP following either a-tDCS or sham stimulation a paired t-test was performed, comparing the magnitude of relative change of MEPs between conditions. For the RPE-clamp test, a one-way repeated measures ANOVA was used to test for statistical differences between the a-tDCS, sham, and control conditions for PO, HR, and VO_2_, respectively. A one-way ANOVA was used to test for statistical differences between the a-tDCS, sham, and control conditions for time to complete the TT, as well as for average PO, average HR, average VO_2_, average RPE, and average PO/RPE ratio. A two-way repeated measures ANOVA (condition × time) was used to test for statistical differences in RPE, PO, and PO/RPEratio for each 25kJ interval of the TT. Post hoc testing was carried out using a Tukey’s post hoc test. With the exception of HR data from the RPE-clamp test, all data were found to be normal distributed using a Shapiro-Wilks test and visual inspection of histograms and QQ-plots. Due to the robustness of the ANOVA test, an ANOVA was used, even when data was not normally distributed. Sphericity of data was tested using the Mauchly’s test. If the assumption of sphericity was violated, the degrees of freedom was corrected using the Greenhouse-Geisser corrections (G-G_cor_). Data are presented as mean ± standard deviation. Statistical significance was accepted at p≤0.05. All calculations were performed in SPSS Version 25.0 (IBM Corp; Armonk, NY, USA).

## Results

### MEPs

**Fig 2:**
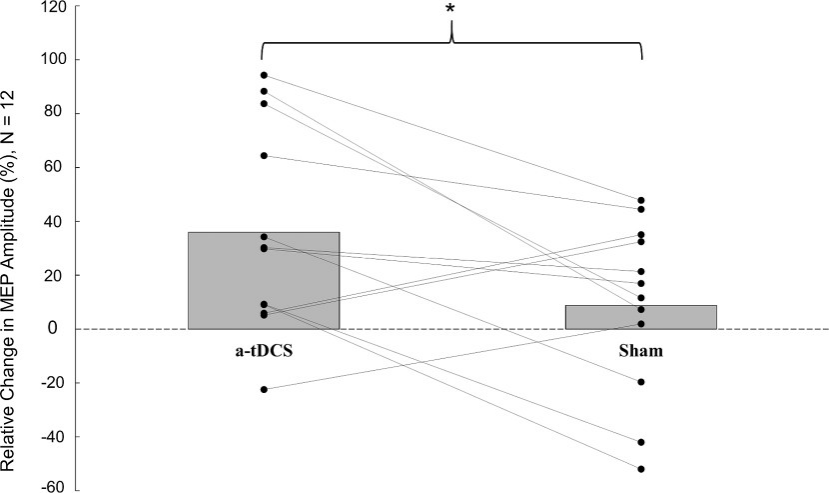
Relative change in peak-to-peak amplitude of motor evoked potentials from pre test to post test during the a-tDCS and sham stimulation protocol. Grey bars represent the group average. Black circles represent individual participants. * denotes significant difference (*P*<0.05). N = 12.

The relative increase in MEPs was significantly larger following a-tDCS (36 ± 36%, pre test = 421.2 ± 336.5 μV, post test = 602.2 ± 599.5 μV) compared to sham stimulation (8.8 ± 31%, pre test = 556.6 ± 420.4 μV, post test = 597.0 ± 497.0 μV) (p = 0.037) (Fig 2).

### RPE-clamp

**Fig 3:**
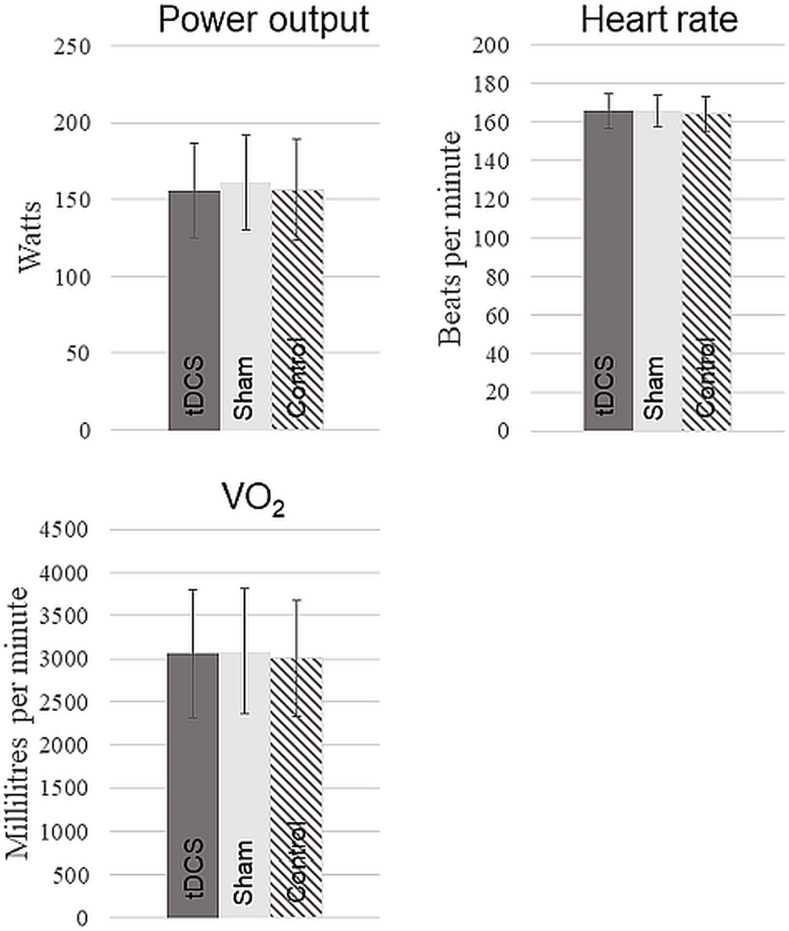
Power output, heart rate, and oxygen consumption (VO_2_) during the RPE-clamp test. The dark grey bars represent the a-tDCS condition, the light grey bars represents the sham condition, and the striped bars represents the control condition. The error bars represent the standard deviation. N = 20.

In the RPE-clamp test, no significant differences were found between a-tDCS, sham, and control conditions in PO (a-tDCS = 156 ± 31 watt, sham = 161 ± 31 watt, control = 156 ± 33 watt, F(2, 38) = 0.828, p = 0.44), HR (a-tDCS = 137 ± 19 bpm, sham = 135 ± 18 bpm, control = 134 ± 17 bpm, F(2, 36) = 0.63, p = 0.54), or VO_2_ (a-tDCS = 2327 ± 542 mL/min, sham = 2350 ± 486 mL/min, control = 2264 ± 493 mL/min, F(2, 38) = 1.09, p = 0.34 (Fig 3).

### Time trial

**Fig 4:**
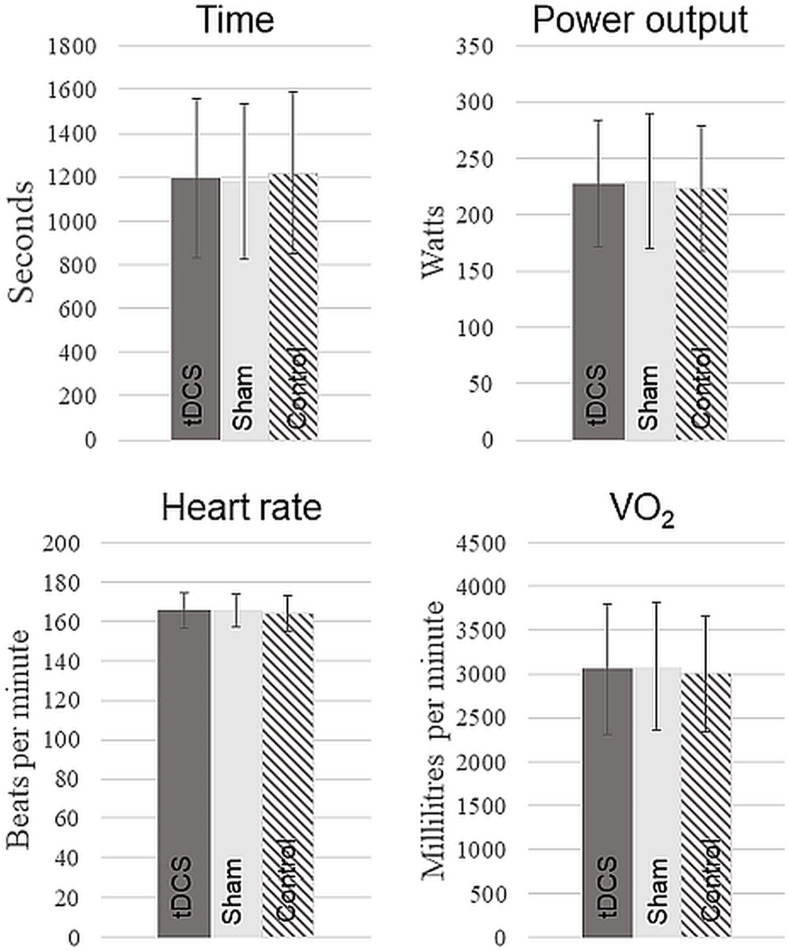
Average time, power output, heart rate, and oxygen consumption (VO_2_) during the 250-kJ time trial. The dark grey bars represent the a-tDCS condition, the light grey bars represents the sham condition, and the striped bars represents the control condition. The error bars represent the standard deviation. N = 20.

The one-way ANOVA revealed no significant differences in the completion time (a-tDCS = 1195 ± 361 s, sham = 1182 ± 355 s, control = 1219 ± 372 s, F(2, 38) = 1.89, p = 0.16), PO (a-tDCS = 227 ± 56 watt, sham = 230 ± 60 watt, control = 223 ± 55 watt, F(2, 38) = 2.05, p = 0.14), HR (a-tDCS = 166 ± 9 bpm, sham = 164 ± 11 bpm, control = 163 ± 11 bpm, F(2, 36) = 0.654, p = 0.52), VO_2_ (a-tDCS = 3058 ± 643 mL/min, sham = 2973 ± 1160 mL/min, control = 3003 ± 557 mL/min, F(2, 38) = 1.40, p = 0.25), RPE (a-tDCS = 16 ± 1, sham = 16 ± 1, control = 16 ± 1, F(2, 38) = 0.441, p = 0.64), or Power/RPE-ratio (a-tDCS = 13.9 ± 3.5, sham = 14.1 ± 3.6, control = 13.7 ± 3.4, F(2, 38) = 2.63, p = 0.08) of the TT (Fig 4).

**Fig 5:**
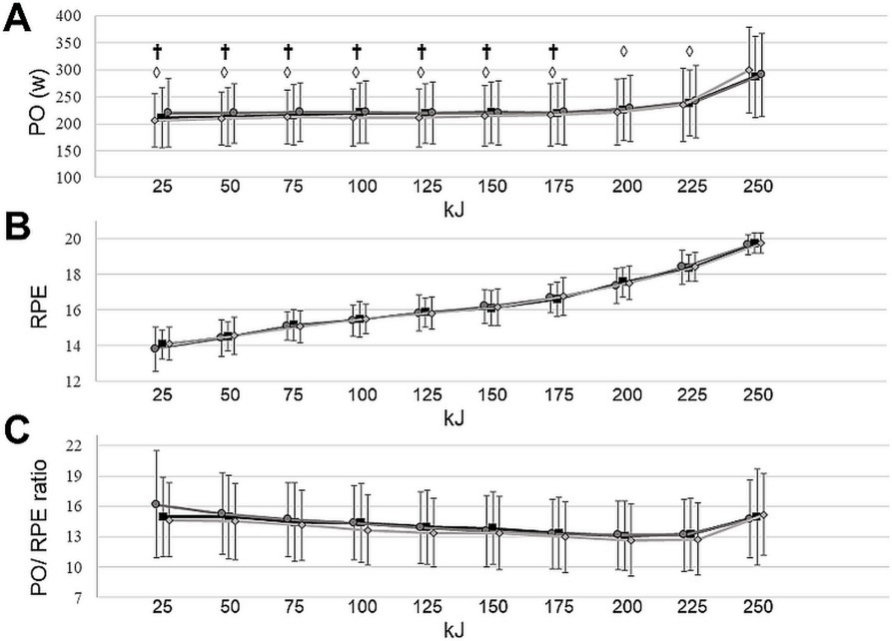
**A:** Average power output (PO) for each 25 kJ interval of the 250-kJ time trial (TT). **B:** Average rate of perceived exertion (RPE) for each 25 kJ interval of the 250-kJ TT. **C:** PO/RPE-ratio for each 25 kJ interval of the 250-kJ TT. The black squares represent the a-tDCS condition, the dark grey circles represents the sham condition, and the light grey diamonds represents the control condition. The error bars represent the standard deviation. † denotes a significant difference from the interval between 200–225 kJ. ◊ denotes a significant difference from the interval between 225-250kJ. N = 20.

Similarly, the two-way repeated measures ANOVA found no significant differences between conditions for the 25kJ intervals with regards to RPE (F(18, 342) = 0.60, p = 0.77) or PO/RPE ratio (F(18, 342) = 1.75, p = 0.13). However, a main effect of time was found for PO (F(9, 171) = 49.96, p = 0.01), with a post hoc showing a higher PO for the interval between 200–225 kJ (238 ± 65 watt) compared to each 25kJ interval between 0–175 kJ (p<0.01) and a higher PO for the 25 kJ interval between 225–250 kJ (292 ± 78 watt) compared to all of the previous intervals (p = 0.01) (Fig 5).

## Discussion

The aim of the present study was to determine the effects of a-tDCS on corticospinal excitability of M1 and self-paced cycling performance during a submaximal RPE-clamp test and a TT. The main finding was a significant increase in corticospinal excitability following a-tDCS. However, this did not lead to any significant differences in PO, HR, or VO_2_ during the RPE-clamp test or the TT.

### Corticospinal excitability

Motor evoked potentials increased significantly following a-tDCS, but not after sham stimulation. The 36% increase in MEPs is in line with findings from previous studies [3,9]. The mechanisms explaining the increased MEPs are not fully understood, but may involve changes in NMDA receptor sensitivity and membrane polarization [26]. Activation of voltage-sensitive Ca^2+^ channels through subthreshold depolarization has been shown in a study performed on rats [27]. The increased MEPs in the present study may therefore be a result of increased sensitivity of NMDA receptors, due to increased influx of Ca^2+^. However, this remains hypothetical, as the present study did not examine these mechanisms directly.

### Cycling performance and regulation of pacing during self-paced exercise

The results of the present study showed that neither of the parameters during the RPE-clamp test and the TT were altered following a-tDCS, despite corticospinal excitability being significantly increased. As RPE has been proposed as a key regulator of pacing during self-paced exercise, a-tDCS was expected to improve performance, as it previously has been shown to downregulate RPE, allowing more work to be produced at the same RPE [9].

In Borg’s model of perceived exertion, both an efferent and afferent component of RPE are acknowledged [20] as the RPE is described as “being an individual’s total physical and psychic reaction to exertion”. The efferent component of the RPE is based on central motor commands generating corollary discharges that project onto the somatosensory cortex [28]. The afferent component refers to a homeostatic afferent pathway identified in the dorsal posterior insula that generates an interoceptive sense of self [29]. The pathway receives inputs such as pain and muscle sensations from mechano- and metabosensitive Group III/IV afferent and provides a conscious awareness of the body’s homeostatic processes [29]. However, the relative contribution of the efferent and the afferent part of the RPE in regulation of pacing during self-paced exercise is still unknown [17]. When considering the results of the present study, a larger contribution of the afferent feedback to the regulation of pacing during self-paced exercise is likely, as the increased corticospinal excitability did not lead to differences in any of the physiological parameters measured during the RPE-clamp test and TT.

The lack of improvements in the TT despite increased corticospinal excitability could be explained by the peripheral governor model by MacIntosh and Shahi [30]. In this model, it is proposed that the regulation of exercise intensity is based on a biological mechanism inside each muscle fiber to suppress the rate by which ATP are used. The peripheral governor are believed to attenuate the cellular activation potentially through decreases in membrane excitability and disturbances in calcium handling [30]. Amann et al. [31] provided evidence for a peripheral governor, as fentanyl injections, which increases the central motor drive, did not lead to a sustained higher PO or improved performance during a 5-km cycling TT. Additionally, *Sidhu et al*. showed using a pharmalogical approach that feedback from group III/IV muscle afferents decreases motor cortex excitability [32]. Such evidence supports the view of MacIntosh and Shahi [30] that central mechanisms may interfere with the pacing during self-paced exercise, but it is the peripheral governor that is the main regulator by limiting the use of ATP in the muscle. A peripheral governor could explain the lack of improvements in the TT despite increased corticospinal excitability in the present study, as the increased motor drive would be overruled by the muscle fibers in order to limit the decline in ATP when replenishment is challenged by the demand.

A significantly higher PO was found in the last two epochs of the TT. This is most likely due to the participants performing a final sprint in an attempt to maximize the result of the test [16]. There were no significant differences between the a-tDCS, sham and control sessions, indicating that this strategy was unaffected by the increased corticospinal excitability obtained in the a-tDCS session.

### Effects of a-tDCS on physiological responses during self-paced exercise

In the present study the HR and VO_2_ responses during the RPE-clamp test and TT did not differ between stimulation conditions. This result is in line with previous studies showing no alterations in cardiovascular or autonomic responses following a-tDCS stimulation of M1 [9,12]. However, it is in contrast to Okano et al. (2015), who showed a significantly reduced HR at submaximal intensities during an incremental cycling task following a-tDCS over the temporal and insular cortex, as a result of increased parasympathetic modulation [11]. As the present study, in line with previous studies [8,9,12], found no effects on HR or VO_2_ consumption following a-tDCS compared to sham stimulation and the control session, this may either indicate that increased excitability of M1 does not influence VO_2_ kinetics directly or that increased excitability of M1 does not influence RPE and power output, and thus no difference is observed in VO_2_. It should be noted however, that several phenomena described in the literature indicates that the central nervous system (CNS) may also serve as a limiting factor, as limitation by peripheral anaerobiosis can not explain that: 1) Less than 100% of muscle fibers are recruited at the end of a task [33], 2) interventions that exclusively alters the CNS, such as administration of acetaminophen, improves performance [34], 3) RPE is a function of relative exercise duration, and not intensity [17], and 4) the end sprint, where increases in pace appears even though this would be where the largest degree of fatigue should be present [35]. This could explain why some studies are able to show a performance enhancing effect of a-tDCS during cycling, while the present study and others are not [14]. The studies that find a positive effect are utilizing TTF cycling protocols, while Barwood et al. (2015) and the present study utilize TT tests, which are fundamentally different.

### Technical considerations and limitations

The coefficient of variation for the PO in the TT in the present study was 3.76%, ranging from 0.29–10.56%. However, performance increases has been shown to be less in TT-tasks, compared to TTF-tasks, as Amann et al. [36] found hypoxia and hyperoxia to affect a TTF-task with 45% and 123%, whereas the effect for the TT was only 5.7% and 4.1%, respectively. Improvements of ∼20–23% in TTF-tasks [8,9,12] may therefore probably not be seen in a TT-task. It is therefore theoretically possible that a small improvement in TT performance could be masked by the inherent variation of 3.76% observed in the present study, although we don’t believe this to be the case.

At the RPE-clamp test, participants were instructed to ride at a constant RPE of 13 on the Borg scale. An RPE of 13 would approximately match a HR of 130 BPM for participants aged 30–50 years [20]. Participants in the present study was on average 26 years, and an RPE of 13 may therefore match a HR being some beats higher than suggested by Borg. An average HR of 135.6 BPM, as in the present study, closely resembles the HR for RPE 13. However, the participants HR ranges from 103–167 BPM, with a low coefficient of variation of 4.16%. This indicates that even though all participants have not been riding with a HR corresponding to an RPE 13, their impression of an RPE 13 was similar throughout all trials.

The data of the present study is based on 16 male and four female subjects, which results in a clear gender imbalance. This imbalance could potentially influence the results of the study. Post hoc analysis in to this matter, however, revealed no such influence. Furthermore, previous studies have not found any gender differences in male and female responses to a-tDCS [37].

In conclusion, a-tDCS administrated over Cz significantly increased corticospinal excitability in the present study. However, this did not lead to a significant increase in power output in the RPE-clamp test, nor did it cause an increase in power output and a decrease in time to completion of the TT. The increased corticospinal excitability observed in the a-tDCS session did not lead to any differences between tDCS, sham and control sessions in terms of PO, HR, VO_2_, or RPE.

### Perspectives

The use on non-invasive brain stimulation as an ergogenic aid in sports performance contexts are quickly becoming popular. While the literature has indeed shown positive effects of this type of stimulation, the performance tests most often utilized, such as time to fatigue tests, are not representative of the demands of real-world sports competitions such as time trials. Our results indicate that a-tDCS, can significantly increase corticospinal excitability as compared to a sham stimulation protocol. This increase in corticospinal excitability, however, have no effect on the performance during a self-paced 10 km cycling time trial or on cycling performance during a submaximal RPE-clamp test. Therefore, although a-tDCS is capable of inducing changes in corticospinal excitability, our results clearly indicate that this does not influence cycling performance in any meaningful way.
